# Supplementary material for: Invitation appeals and STEM academic scientists research participation: Findings from six survey experiments
Source: PLoS One. 2025 Jun 17;20(6):e0326331. doi: 10.1371/journal.pone.0326331 (PMC12173187; doi:10.1371/journal.pone.0326331)
Supplement: S1 Table — (PDF) [file pone.0326331.s001.pdf]

S1 Table. Number of Randomly Selected Institutions for Sampling Scientists.

Fields	Number of randomly selected R1 institutions	Number of all R1 institutions
Biology	106	131
Civil and environmental engineering	46	131
Geography	46	131
Public health	61	61
