# Supplementary material for: Invitation appeals and STEM academic scientists research participation: Findings from six survey experiments
Source: PLoS One. 2025 Jun 17;20(6):e0326331. doi: 10.1371/journal.pone.0326331 (PMC12173187; doi:10.1371/journal.pone.0326331)
Supplement: S2 Table — (PDF) [file pone.0326331.s008.pdf]

S2 Table. Balance Test Results for Information Appeal Experiments.

	Survey of Scientists' Perceptions of Surveys			Vaccine Survey		
Treatment Conditions	No Information Appeal (N[a]=131; No. of words[b]=179; FK Level[c]=11.7)	Some Information Appeal (N=132; No. of words=232; FK Level=13)	Much Information Appeal (N=135; No. of words=277; FK Level=12.7)	No Information Appeal (N=276; No. of words=169; FK Level=11.7)	Some Information Appeal (N=277; No. of words=211; FK Level=12.3)	Much Information Appeal (N=278; No. of words=255; FK Level=13.1)
Female	14.6	14.6	17.7	16.4	15.3	18.3
Field						
Biology	14.8	15.4	15.1	22.3	21.8	20.6
Civil and Environmental Engineering	5.2	4.2	4.9	–	–	–
Geography	2.1	3.4	2.9	–	–	–
Public Health	11.2	10.4	10.4	11.0	11.6	12.9
Rank						
Full Professor	12.9	12.9	13.9	14.0	13.4	13.1
Associate Professor	7.9	8.4	5.8	6.9	6.7	7.2
Assistant Professor	6.6	7.9	8.4	6.7	6.8	7.3
Non-tenure Track Researcher	5.5	4.5	5.3	5.4	6.6	6.0
Have been invited to previous SciOPS survey	–	–	–	26.7	26.2	26.5

[a]N indicates the size of eligible samples that exclude ineligible scientists (i.e., deceased, retired, or no longer in academia) and those unreachable during the survey administration period (i.e., rotations, or short-term leaves and out of office).
[b]No. of words indicate the number of words in an invitation email.
[c]FK level indicates the Flesch-Kincaid Grade Level, which shows the required U.S. grade level of education to be able to understand the text of the invitation emails. A score of 9 means that a ninth grader can understand the survey invitation email.
Proportions (%) of the sample are reported.
