# Supplementary material for: Invitation appeals and STEM academic scientists research participation: Findings from six survey experiments
Source: PLoS One. 2025 Jun 17;20(6):e0326331. doi: 10.1371/journal.pone.0326331 (PMC12173187; doi:10.1371/journal.pone.0326331)
Supplement: S3 Table — (PDF) [file pone.0326331.s009.pdf]

S3 Table. Balance Test Results for Representation Appeal Experiments.

Treatment Conditions	COVID-19 Survey Wave 2		Public Trust on Science Survey		Women's Health Survey	
	Self-representation Appeal (N[a]=951; No. of words[b] =222; FK Level[c]=10)	Community-representation Appeal (N=921; No. of words =224; FK Level=10.8)	Self-representation Appeal (N=1263; No. of words=266; FK Level=10.8)	Community-representation Appeal (N=1273; No. of words=263; FK Level=11.6)	Self-representation Appeal (N=200; No. of words =169; FK Level=10.6)	Community-representation Appeal (N=200; No. of words =172; FK Level=11.3)
Female	16.0	15.1	22.4	23.0	22.2	23.8
Field						
Biology	38.0	35.7	17.6	16.3	22.2	22.8
Civil and Environmental Engineering	12.8	13.5	–	–	9.8	8.2
Geography	–	–	–	–	5.8	4.5
Public Health	–	–	0.322	0.339	12.2	14.5
Rank						
Full Professor	21.5	21.0	19.1	18.3	16.8	22.9
Associate Professor	11.5	10.7	11.5	12.3	12.2	10.7
Assistant Professor	12.0	12.2	10.8	12.6	9.4	8.9
Non-tenure Track Researcher	5.7	5.3	8.3	6.9	11.7	7.4

[a]N indicates the size of eligible samples that exclude ineligible scientists (i.e., deceased, retired, or no longer in academia) and those unreachable during the survey administration period (i.e., rotations, or short-term leaves and out of office).
[b]No. of words indicate the number of words in an invitation email.
[c]FK level indicates the Flesch-Kincaid Grade Level, which shows the required U.S. grade level of education to be able to understand the text of the invitation emails. A score of 9 means that a ninth grader can understand the survey invitation email.
Proportions (%) of the sample are reported.
