# Supplementary material for: Invitation appeals and STEM academic scientists research participation: Findings from six survey experiments
Source: PLoS One. 2025 Jun 17;20(6):e0326331. doi: 10.1371/journal.pone.0326331 (PMC12173187; doi:10.1371/journal.pone.0326331)
Supplement: S4 Table — (PDF) [file pone.0326331.s010.pdf]

S4 Table. Balance Tests Results for COVID-19 Survey Wave 4.

	Self-representation Appeal		Community-representation Appeal	
Treatment Conditions	No Information Appeal (N=443; No. of words=118; FK Level=9.9)	Some Information Appeal (N=438; No. of words=126; FK Level=10.2)	No Information Appeal (N=436; No. of words=195; FK Level=10.9)	Some Information Appeal (N=438; No. of words=186; FK Level=10.8)
Female	7.9	8.3	7.3	7.2
Field				
Biology	19.5	18.1	17.9	18.6
Civil and Environmental Engineering	5.8	6.8	7.0	6.3
Rank				
Full Professor	10.8	11.0	10.4	11.2
Associate Professor	5.7	5.9	5.1	5.8
Assistant Professor	5.9	5.0	6.5	6.0
Non-tenure Track Researcher	2.8	3.0	3.0	2.0
SciOPS panel member	1.5	1.5	1.6	1.5

[a]*N indicates the size of eligible samples that exclude ineligible scientists (i.e., deceased, retired, or no longer in academia) and those unreachable during the survey administration period (i.e., rotations, or short-term leaves and out of office).*
[b]*No. of words indicate the number of words in an invitation email.*
[c]*FK level indicates the Flesch-Kincaid Grade Level, which shows the required U.S. grade level of education to be able to understand the text of the invitation emails. A score of 9 means that a ninth grader can understand the survey invitation email.*
Proportions (%) of the sample are reported.
Standard deviations are in parentheses.
