# Supplementary material for: Invitation appeals and STEM academic scientists research participation: Findings from six survey experiments
Source: PLoS One. 2025 Jun 17;20(6):e0326331. doi: 10.1371/journal.pone.0326331 (PMC12173187; doi:10.1371/journal.pone.0326331)
Supplement: S5 Table — (PDF) [file pone.0326331.s011.pdf]

**S5 Table. Logit Models Results of Information Appeal Experiment.**

| | Model 1: Survey of Scientists' Perceptions of Surveys | Model 2: Vaccine Survey | Model 3: COVID-19 Survey Wave 4 |
|---|---|---|---|
| Some information condition | -0.096*<br>(0.053) | -0.030<br>(0.042) | 0.028**<br>(0.013) |
| Much information condition | -0.174***<br>(0.052) | 0.0442<br>(0.042) | |
| Female | 0.033<br>(0.051) | 0.0170<br>(0.035) | 0.006<br>(0.015) |
| Civil and environmental engineering | -0.085<br>(0.069) | | -0.012<br>(0.015) |
| Geography | 0.029<br>(0.092) | | |
| Public health | 0.011<br>(0.056) | -0.005<br>(0.036) | |
| Assistant professor | -0.047<br>(0.079) | -0.025<br>(0.054) | 0.0224<br>(0.028) |
| Associate professor | 0.011<br>(0.081) | -0.000<br>(0.055) | -0.006<br>(0.025) |
| Full professor | 0.113<br>(0.075) | 0.0315<br>(0.049) | 0.010<br>(0.024) |
| Have been invited to SciOPS survey | | -0.118***<br>(0.043) | |
| Representation appeal: Community-representation condition | | | -0.004<br>(0.013) |
| SciOPS panel member (1=Yes) | | | 0.315***<br>(0.048) |
| N | 380 | 822 | 1755 |
| McFadden's R2 | 0.037 | 0.005 | 0.077 |

*Average marginal effects are reported.*
**p<0.1, **p<0.05, ***p<0.01.*
*Standard errors are reported in parentheses.*
*Reference group: No information - experimental conditions, Biology - field, Non-tenure track researcher - rank, and Self-representation - experimental conditions.*
