# Supplementary material for: Invitation appeals and STEM academic scientists research participation: Findings from six survey experiments
Source: PLoS One. 2025 Jun 17;20(6):e0326331. doi: 10.1371/journal.pone.0326331 (PMC12173187; doi:10.1371/journal.pone.0326331)
Supplement: S6 Table — (PDF) [file pone.0326331.s012.pdf]

S6 Table. Logit Models Results of Representation Appeal Experiment.

	Model 4: COVI-19 Survey Wave 2	Model 5: Public Trust on Science Survey	Model 6: Women's Health Survey	Model 7: COVID-19 Survey Wave 4
Representation Appeal Condition: Community-representation Condition	-0.021<br>(0.017)	0.002<br>(0.017)	-0.097**<br>(0.048)	-0.004<br>(0.013)
Female	0.066***<br>(0.020)	-0.006<br>(0.017)	0.112**<br>(0.050)	0.006<br>(0.015)
Civil and environmental engineering	0.017<br>(0.019)		-0.048<br>(0.066)	-0.012<br>(0.015)
Geography			-0.061<br>(0.077)	
Public health		0.047***<br>(0.018)	-0.054<br>(0.056)	
Chemistry				
Computer and Information Science and Engineering				
Assistant professor	0.125***<br>(0.045)	-0.001<br>(0.027)	-0.169***<br>(0.063)	0.0224<br>(0.028)
Associate professor	0.081*<br>(0.044)	0.002<br>(0.027)	-0.107<br>(0.065)	-0.006<br>(0.025)
Full professor	0.092**<br>(0.037)	0.008<br>(0.026)	-0.053<br>(0.064)	0.010<br>(0.024)
Information appeal: Some information				0.028**<br>(0.013)
SciOPS panel member (1=Yes)				0.315***<br>(0.048)
N	1872	2531	393	1755
McFadden's R^2	0.016	0.003	0.034	0.077

Average marginal effects are reported.
**p<0.1, **p<0.05, ***p<0.01.*
Standard errors are reported in parentheses.
Reference group: Self-representation - experimental conditions, Biology - field, Non-tenure track researcher - rank, and No information - experimental conditions.
