# Supplementary material for: Invitation appeals and STEM academic scientists research participation: Findings from six survey experiments
Source: PLoS One. 2025 Jun 17;20(6):e0326331. doi: 10.1371/journal.pone.0326331 (PMC12173187; doi:10.1371/journal.pone.0326331)
Supplement: S7 Table — (PDF) [file pone.0326331.s013.pdf]

S7 Table. Field-specific Sensitivity Check for Vaccine Survey and Women's Health Survey

	Fields	No information condition	Some information condition	Much information condition	No information vs Some information difference	Some information vs Much information	No information vs Much information
Vaccine Survey	Public Health	0.308	0.354	0.458	0.046	0.104	0.150*
	Biology	0.411	0.337	0.398	-0.074	0.061	-0.013
		Self-representation Condition	Community-representation Condition	Response rate difference			
Women's Health Survey	Public Health	0.408	0.276	-0.132	–	–	–
	Biology	0.438	0.352	-0.086	–	–	–
	Geography	0.391	0.222	-0.169	–	–	–
	Civil and Environmental Engineering	0.308	0.273	-0.035	–	–	–

**p<0.1, **p<0.05, ***p<0.01.*
