# Supplementary material for: Invitation appeals and STEM academic scientists research participation: Findings from six survey experiments
Source: PLoS One. 2025 Jun 17;20(6):e0326331. doi: 10.1371/journal.pone.0326331 (PMC12173187; doi:10.1371/journal.pone.0326331)
Supplement: S1 Appendix — (PDF) [file pone.0326331.s002.pdf]

S1 Appendix. Invitation Emails Template for Survey of Scientists' Perceptions of Surveys (Information Appeal Experiment).

Subject Line: SciOPS panelists survey practices

Dear Dr. [Surname only]:

Thank you for joining SciOPS (Scientist Opinion Panel Survey), a nationally representative panel of the Science, Technology and Innovation (STI) community.

I am writing to ask for your help with a national survey of the STI community concerning **researchers' survey practices**. We are interested in your opinions about our survey methods!

[Randomized (1) no information appeal: " "/

(2) some information appeal:"This particular survey will ask about your personal survey experiences, your opinion regarding important topics for future study and related issues. It will help us in the future to better design and focus SciOPS for communicating your opinions and experiences to the scientific community, policymakers, journalists and other stakeholders."

(3)much information appeal:"SciOPS is an important tool for engaging the scientific community and ensuring their voices are represented in the policy-making process. It also seeks to monitor trends over time and communicate to the public the importance and value to society of scientific research. It does so using a rigorous, replicable and transparent survey process. This particular survey will ask about your personal survey experiences and opinions regarding them. It will help us in the future to better design and focus SciOPS for communicating your opinions and experiences to the public, policymakers, journalists and other stakeholders."]

The questionnaire is short and should only take about ten minutes of your time.

Please access the online survey by entering the following URL into your browser and using the provided username and password.

You can follow this link to the Survey: [insert link]

Username: [insert unique user ID]
Password: [insert password]

The survey is confidential and your participation is voluntary.

We will present the aggregate findings on our SciOPS (Scientist Opinion Panel Survey) science communication platform, which aims to inform the science community, policymakers and other stakeholders about this critical issue.

Thank you for considering this request. I know you are extremely busy and sincerely appreciate the time you take to respond to the survey. If you have any questions or concerns, please contact me at xxxxx@xx.edu.

Kind regards,
XXX, PhD, Director
Center for XXXXX
XXXX University
