# Supplementary material for: Invitation appeals and STEM academic scientists research participation: Findings from six survey experiments
Source: PLoS One. 2025 Jun 17;20(6):e0326331. doi: 10.1371/journal.pone.0326331 (PMC12173187; doi:10.1371/journal.pone.0326331)
Supplement: S2 Appendix — (PDF) [file pone.0326331.s003.pdf]

S2 Appendix. Invitation Emails Template for Vaccine Survey (Information Appeal Experiment).

Subject Line: SciOPS Survey Invitation – Vaccines and Public Policy

Dear Dr. [Surname only]:

I am writing to ask for your help with a national survey of the STI community concerning **the role of scientists in communicating and developing public policy about vaccines** that is being conducted by the SciOPS (Scientist Opinion Panel Survey) team.

[Randomized (1) no information appeal: " " /

(2) some information appeal: "The survey will ask you about your personal opinions regarding vaccines, vaccine hesitancy, and relevant public policy. Your responses will help us better understand how scientists and the scientific community view these important questions and help inform public debate on these topics." /

(3) much information appeal: "There is currently considerable public interest and debate about this topic! The survey will ask you about your personal opinions and beliefs regarding vaccines, vaccine hesitancy and vaccine messaging, the effectiveness of relevant public policy tools, and your institution's response to recent vaccine controversies. Your responses will help us better understand how scientists and the scientific community view these important questions and how the opinions of the scientific community compare to those of the general population. Findings will also help inform public debate on these topics."]

The questionnaire is short and should only take about ten minutes of your time.

Please access the online survey by entering the following URL into your browser and using the provided username and password.

You can follow this link to the Survey: [insert link]

Username: [insert unique user ID]
Password: [insert password]

The survey is confidential and your participation is voluntary.

We will present the aggregate findings with other researchers and on our SciOPS science communication platform, which aims to inform the science community, policymakers and other stakeholders about this critical issue.

Thank you for considering this request. I know you are extremely busy and sincerely appreciate the time you take to respond to the survey. If you have any questions or concerns, please contact me at xxxxx@xx.edu

Kind regards,
XXXX, PhD, Director
Center for XXXXXXXX
XXXXX University
