# Supplementary material for: Invitation appeals and STEM academic scientists research participation: Findings from six survey experiments
Source: PLoS One. 2025 Jun 17;20(6):e0326331. doi: 10.1371/journal.pone.0326331 (PMC12173187; doi:10.1371/journal.pone.0326331)
Supplement: S3 Appendix — (PDF) [file pone.0326331.s004.pdf]

S3 Appendix. Invitation Emails Template for COVID-19 Wave 2 Survey (Representation Appeal Experiment).

Subject Line: How has COVID-19 affected [Randomized (1) self-representation appeal: "your" / (2): community-representation appeal: "academic "] research?

Dear Dr. [Surname only]:

I am writing to ask for your help with a national survey of scientists and engineers concerning the effects of COVID-19 on [Randomized (1) self-representation appeal: "your personal research. You are part of a random sample that has been chosen to complete a brief questionnaire. Your opinion and experiences are needed to inform further understanding about this important topic." / (2) community-representation appeal: "academic research. You are part of a random sample that has been chosen to represent the scientific community by completing a brief questionnaire concerning this important topic."]

As you know, the COVID-19 has affected most areas of life in the US. Your response is vitally important for learning about the experience of scientists such as yourself. The survey will ask you about COVID-19 impacts on your research and funding, as well as a few other topics.

The questionnaire is short and should take no more than ten minutes of your time.

Please access the online survey by entering the following URL into your browser and using the provided username and password.

You can follow this link to the Survey: [insert link]

Username: [insert unique user ID]
Password: [insert password]

The survey is confidential and your participation is voluntary.

We will present the aggregate findings on our SciOPS (Scientist Opinion Panel Survey) science communication platform, which aims to inform the science community, policymakers and other stakeholders about this critical issue.

Thank you for considering this request. I know you are extremely busy and sincerely appreciate the time you take to respond to the survey. If you have any questions or concerns, please contact me at xxxxxxx@xx.edu

Kind regards,
XXX, PhD, Director
Center for XXXXXXXXX
XXXXX University
