# Supplementary material for: Invitation appeals and STEM academic scientists research participation: Findings from six survey experiments
Source: PLoS One. 2025 Jun 17;20(6):e0326331. doi: 10.1371/journal.pone.0326331 (PMC12173187; doi:10.1371/journal.pone.0326331)
Supplement: S4 Appendix — (PDF) [file pone.0326331.s005.pdf]

S4 Appendix. Invitation Emails Template for Public Trust Survey (Representation Appeal Experiment).

Subject Line: Survey [Randomized (1) self-representation appeal:“regarding your views” / (2) community-representation appeal: “ ”) on public trust in science

Dear Dr. [Surname only]:

I am writing to ask for your help with a national survey of scientists and engineers concerning the [Randomized (1) self-representation appeal: “your personal view on factors that influence the public’s trust in science. You are part of a random sample that has been chosen to complete a brief questionnaire. Your opinion and experiences are needed to inform further understanding about this important topic.” / (2) community-representation appeal: “scientific community’s views on factors that influence public trust in science. You are part of a random sample that has been chosen to represent the scientific community by completing a brief questionnaire concerning this important topic.”]

As you know, due to the pandemic and election this past year, public trust in science and scientists has been an area of intense debate. Your response is vitally important for learning about the experience of members of the scientific community such as yourself. This survey will ask about your perspectives on public trust in science, including factors that diminish trust, ways to increase trust, and who should be responsible for preserving the public’s trust, as well as a few other topics.

The questionnaire is short and should take no more than ten minutes to complete.

Please access the online survey by entering the following URL into your browser and using the provided username and password.

You can follow this link to the Survey: [insert link]

Username: [insert unique user ID]
Password: [insert password]

The survey is confidential and participation is voluntary.

We will present the aggregate findings on our SciOPS (Scientist Opinion Panel Survey) science communication platform, which aims to inform the science community, policymakers and other stakeholders about this critical issue.

Thank you for considering this request. I know you are extremely busy and sincerely appreciate the time you take to respond to the survey. If you have any questions or concerns, please contact me at xxxxxx@xx.edu.

Kind regards,
XXX, PhD, Director
Center for XXXXXXXX
XXXXX University
