# Supplementary material for: Invitation appeals and STEM academic scientists research participation: Findings from six survey experiments
Source: PLoS One. 2025 Jun 17;20(6):e0326331. doi: 10.1371/journal.pone.0326331 (PMC12173187; doi:10.1371/journal.pone.0326331)
Supplement: S5 Appendix — (PDF) [file pone.0326331.s006.pdf]

S5 Appendix. Invitation Emails Template for Women's Health Survey (Representation Appeal Experiment).

Subject Line: [Randomized (1) self-representation appeal: "Your" / (2): community-representation appeal: "Scientific Community "] views on women's reproductive healthcare

Dear Dr. [Surname only]:

I am writing to ask for your help with a national survey of scientists and engineers concerning [Randomized (1) self-representation appeal:"your personal views on women's reproductive healthcare. You are part of a random sample that has been chosen to complete a brief questionnaire concerning this important topic." / (2) community-representation appeal:"women's reproductive healthcare. You are part of a random sample that has been chosen to represent the scientific community by completing a brief questionnaire concerning this important topic."]

The questionnaire is short and should take no more than ten minutes to complete.

Please access the online survey by entering the following URL into your browser and using the provided username and password.

You can follow this link to the Survey: [insert link]

Username: [insert unique user ID]
Password: [insert password]

The survey is confidential and participation is voluntary.

We will present the aggregate findings on our SciOPS (Scientist Opinion Panel Survey) science communication platform, which aims to inform the science community, policymakers and other stakeholders about this critical issue.

Thank you for considering this request. I know you are extremely busy and sincerely appreciate the time you take to respond to the survey. If you have any questions or concerns, please contact me at xxxxx@xx.edu

Kind regards,
XXXX, PhD, Director
Center for XXXXXXX
XXX University
