# Supplementary material for: Invitation appeals and STEM academic scientists research participation: Findings from six survey experiments
Source: PLoS One. 2025 Jun 17;20(6):e0326331. doi: 10.1371/journal.pone.0326331 (PMC12173187; doi:10.1371/journal.pone.0326331)
Supplement: S6 Appendix — (PDF) [file pone.0326331.s007.pdf]

S6 Appendix. Invitation Emails Template for COVID-19 Wave 4 Survey (Two by Two - Information and Representation Appeals Experiment).

Subject Line: Long-Term Impact of COVID-19 on [Randomized (1) self-representation appeal "Your" / (2): community-representation appeal: "Scientific"] Research

Dear Dr. [Surname only],

How did COVID-19 [Randomized (1) self-representation appeal: "**impact your research**?" / (2) community-representation appeal: "**impact scientific research in the US**"] Please help answer this question by completing a very brief – less than 10 minutes – questionnaire to share [Randomized (1) self-representation appeal: "your personal pandemic experience" / (2) community-representation appeal: "pandemic experience"].

[Randomized (A) no information appeal: "" /

(B) some information appeal: "As you know, multiple studies have documented the numerous immediate effects of the pandemic on scientists like you. Less attention has been given to the longer-term impacts, the topic that we focus on here and are requesting your help with. In particular, we are interested in your general reflections on the ways that your COVID-19 experiences may have influenced your funding, research, and overall productivity over the past four years."]

You can access the online questionnaire by entering the following unique URL into your browser.

Please follow this unique link to the Questionnaire: [insert link]

As always, your participation is voluntary and all responses are strictly confidential. We will make the findings available on our SciOPS (Scientist Opinion Panel Survey) website and notify you when they are available.

Thank you for considering this request. I know you are extremely busy and sincerely appreciate the time you take to respond. If you have any questions or concerns, please contact me at xxxxxxxx@xx.edu.

Kind regards,
XXX, PhD, Director
Center for XXXXXXXXXX
XXXXX University
