# Supplementary material for: Invitation appeals and STEM academic scientists research participation: Findings from six survey experiments
Source: PLoS One. 2025 Jun 17;20(6):e0326331. doi: 10.1371/journal.pone.0326331 (PMC12173187; doi:10.1371/journal.pone.0326331)
Supplement: Attachment — Submitted filename: Review PLOS.docx [file pone.0326331.s014.docx]

The manuscript presents a timely and methodologically rigorous investigation into the effectiveness of different invitation appeals (information and representation) on survey response rates among STEM academic scientists. The topic is highly relevant given the challenges of declining response rates and the politicization of science. However, several issues require clarification or revision to strengthen the manuscript.

**Major Comments**

Theoretical Framework and Hypotheses

The application of leverage-salience theory is appropriate but could be more explicitly tied to the experimental design. How do the authors operationalize "salience" in the context of STEM scientists’ survey participation?

Hypothesis 1 assume that less detailed invitations will yield higher response rates, but the results are mixed. The discussion should more thoroughly reconcile these findings with the theory (e.g., by addressing how topic polarization might moderate salience).

Mixed Findings and Interpretation

The inconsistent results for both information and representation appeals are a strength (reflecting real-world complexity) but also a limitation. The authors should more explicitly discuss why certain appeals worked in specific contexts (e.g., polarized topics like women’s health vs. less salient topics).

Subgroup analyses (e.g., by career stage) are insightful but could be better integrated into the theoretical discussion. For example, why might non-tenure-track researchers respond more to self-representation appeals?

Generalizability

The sample is limited to U.S. R1 universities and six STEM fields. The authors should clarify whether findings might differ for non-STEM academics, industry scientists, or international contexts.

The reliance on SciOPS panel members (a self-selected group) may introduce bias. How might this affect the external validity of the results?

Methodological Transparency

The randomization process is well-described, but the manuscript would benefit from including the exact wording of all appeal conditions in the main text (not just supplemental materials).

Response rates varied widely across surveys (e.g., 7.9% in COVID-19 Wave 4 vs. 44.3% in the Scientists’ Perceptions survey). Were there differences in survey length, timing, or incentives that could explain this?

Practical Implications

The recommendations for tailoring appeals are useful but somewhat generic. Could the authors provide more specific guidelines (e.g., "For polarized topics, use self-representation appeals with mid-level detail")?

The finding that prior interaction with SciOPS diminishes the need for crafted appeals is intriguing. Is this a trust effect or familiarity effect? This could be explored further.

**Minor Comments**

Clarity and Structure

The abstract could better highlight the mixed findings upfront (e.g., "Results show context-dependent effects...").

Table 2 and 3 are clear, but the significance markers (e.g., *p<0.1) could be better explained in the captions. Check for consistency in reporting p-values (e.g., p<0.1 vs. p<0.05) across tables.

**Limitations**

The authors note that some nonrespondents may not have read the email content, but they could also discuss whether subject lines (uncontrolled in the experiments) might have influenced open rates.

The potential for topic interest to confound results is acknowledged, but could be addressed statistically (e.g., by controlling for field-specific response rates).

Ethics and Data Availability

Ethical approval is noted, but the consent process (implied via email/survey initiation) could be briefly justified given the lack of written consent.

Data availability is restricted due to privacy concerns, but could the authors share de-identified data or a codebook?

Improve readability by streamlining the methods section and adding a visual summary of key findings (e.g., a conceptual diagram).

Ensure all supplemental materials (e.g., S1 Appendix) are cited in the main text.
