# Supplementary material for: Invitation appeals and STEM academic scientists research participation: Findings from six survey experiments
Source: PLoS One. 2025 Jun 17;20(6):e0326331. doi: 10.1371/journal.pone.0326331 (PMC12173187; doi:10.1371/journal.pone.0326331)
Supplement: Attachment — Submitted filename: Response to Reviewers.docx [file pone.0326331.s016.docx]

**Reviewer #1**

The manuscript presents a timely and methodologically rigorous investigation into the effectiveness of different invitation appeals (information and representation) on survey response rates among STEM academic scientists. The topic is highly relevant given the challenges of declining response rates and the politicization of science. However, several issues require clarification or revision to strengthen the manuscript.

Response: We appreciate your comments and address your concerns point-by-point.

**Major Comments**

Theoretical Framework and Hypotheses

The application of leverage-salience theory is appropriate but could be more explicitly tied to the experimental design. How do the authors operationalize "salience" in the context of STEM scientists’ survey participation?

Response: Thank you very much for raising this important point. In this study, salience is not treated as a separate measured variable but rather serves as the theoretical foundation for developing our hypotheses and experimental design. Grounded in leverage-salience theory, we conceptualize salience as the extent to which specific features of a survey invitation motivate individuals to respond. Individuals evaluate and leverage multiple attributes of a survey invitation—such as monetary incentives, or topic—when deciding whether to participate, and those attributes perceived as important are considered “salient.” If a particular appeal leads to a significantly higher response rate, we interpret it as more salient for a given population.

To operationalize and evaluate salience in the context of our experiment, we integrate it within the design of information and representation appeals. Specifically, we operationalize information salience by comparing detailed versus brief amounts of information, and representation salience by comparing self-representation versus community-representation appeals. We have clarified this theoretical rationale and its connection to our experimental design in the theory section (lines 197–198, 204–207, and 222–224).

Hypothesis 1 assume that less detailed invitations will yield higher response rates, but the results are mixed. The discussion should more thoroughly reconcile these findings with the theory (e.g., by addressing how topic polarization might moderate salience).

Response: Thank you very much for the comments on mixed findings regarding hypothesis 1. To reconcile the results from our information appeal experiment, we draw on the Elaboration Likelihood Model, which explains how individuals are persuaded through either a central or peripheral route, depending on their motivation to engage with the issue and their ability to process information. This theoretical framework supports our interpretation that the salience of detailed versus concise information varies according to the relevance of the survey topic and the characteristics of the recipients. Please see our revised discussion of the information appeal findings in the revised manuscript (lines 446–479, 490-499, and 505-507).

Mixed Findings and Interpretation

The inconsistent results for both information and representation appeals are a strength (reflecting real-world complexity) but also a limitation. The authors should more explicitly discuss why certain appeals worked in specific contexts (e.g., polarized topics like women’s health vs. less salient topics).

Response: We appreciate your comments on mixed findings and their interpretation, which is taken seriously with care. We have rewritten much of the findings discussion for both information and representation appeal experiments to better reconcile the mixed results by articulating the various theoretical rationales behind them, particularly in relation to survey topic, respondents’ career stage, and their prior experience with SciOPS.

Our revisions consist of two key theoretical integrations. First, we draw on the elaboration likelihood model to explain how STEM academic scientists respond differently to detailed versus concise information. The effectiveness of detailed information depends on the relevance of the survey topic to the STEM academic scientific community, as well as respondents’ motivation and capacity to process information, both of which are shaped by their career stage and familiarity with SciOPS.

Second, we incorporate self-categorization theory to explain how the effectiveness of representation appeals are contingent on whether respondents prioritize personal identity over group identity in the context of identity threat. Specifically, when responding to contentious survey topics in a polarized environment, respondents with weaker group identity, i.e., non-tenure-track scientists, may prefer self-representation over community-representation when the group faces potential threats. Please see our elaboration about the connections between self-categorization theory with our findings at our response to the next comment below.

Together, these two theoretical frameworks provide a stronger foundation for interpreting the mixed findings. They not only enhance the generalizability of our results but also offer important practical implications for survey administrators, underscoring the need to design survey messaging based on recipients’ information processing capacity, self-identity and the relevance of the survey topic.

Subgroup analyses (e.g., by career stage) are insightful but could be better integrated into the theoretical discussion. For example, why might non-tenure-track researchers respond more to self-representation appeals?

Response: Thank you for your comments on the subgroup analyses. As we discussed above, we explain that the salience of detailed information is contingent on STEM academic scientists’ career stage using the elaboration likelihood model. In brief, scientists at different stages of their careers have varying levels of time and cognitive capacity to process persuasive messages, leading them to rely on either the central or peripheral route of information processing when evaluating survey requests.

We also agree that integrating theory is important to understand why non-tenure-track researchers respond more to self-representation appeals in the Women’s Health Survey. To explain this result, we draw on self-categorization theory, which suggests that individuals with weaker group identification are more likely to disidentify from the group and prioritize their personal identity when the group faces external threats. Non-tenure-track researchers, who often experience institutional marginalization and identity insecurity within the academic scientific community, may be more inclined to prioritize self-protection over group affiliation. This tendency is likely enhanced in polarized environments where the broader STEM academic scientific community is under potential threats of public scrutiny. We elaborate on this interpretation in the discussion section of the representation appeal experiment (lines 550–560).

Generalizability

The sample is limited to U.S. R1 universities and six STEM fields. The authors should clarify whether findings might differ for non-STEM academics, industry scientists, or international contexts.

Response: Thank you for highlighting the importance of generalizability. In the original manuscript, we noted this as a limitation in the final sentence of the limitations section. We agree that this point warrants clearer emphasis and appreciate the opportunity to strengthen our discussion. In our revision, we have now addressed this limitation more explicitly into the future research section (lines 671–680). We acknowledge that our findings are based on researchers in four STEM fields at U.S. R1 universities and may not generalize to non-STEM academics, industry scientists, or international scholars. These groups may differ in institutional pressures, research norms, and time availability, all of which could influence their responses to survey appeals. We recommend that future studies explore whether and how the effects of information and representation appeals vary across broader academic communities and other populations. We also discuss how our findings may inform future research targeting non-academic populations, thereby extending the implications of our work beyond the academic community, contributing to survey methodology more broadly, and increasing its relevance for a wider audience.

The reliance on SciOPS panel members (a self-selected group) may introduce bias. How might this affect the external validity of the results?

Response: Thank you for this observation. We agree that SciOPS panel members self-selected into the SciOPS panel, likely due to their prosocial orientation and commitment to science communication. As we now note in our response to the comments regarding survey response rates (see below), SciOPS panel members did in fact exhibit higher response rates than non-panel participants.

Importantly, we are able to include both SciOPS panel members and non-panel participants in an experiment. As discussed in our subgroup analysis of the COVID-19 Survey Wave 4 (lines 500–510), we find heterogeneity in how different groups respond to information appeals. Specifically, SciOPS panel members place greater value on receiving topic-relevant information in the survey invitation than do non-panel members, reflecting a meaningful variation within the academic science community rather than bias from self-selection. This variation suggests that commitment to science communication can shape how information appeals are processed and remains externally valid within the STEM academic population. This observed heterogeneity further reinforces the importance of designing effective survey outreach strategies to account for respondent characteristics and subgroup differences. We highlight this in the discussion section (line 593-601).

However, beyond this, their behaviors and opinions are broadly representative of the scientific community from which they are drawn. The SciOPS survey panel is designed to in general understand the behaviors and attitudes of the targeted population––U.S.-based STEM academic scientists. SciOPS panel members are randomly sampled from the targeted population and subsequently recruited to participate in ongoing surveys. Using a survey panel design enhances response rates (i.e., a lower response rate introduces nonresponse bias, which also threatens external validity) and allows for the long-term observation of behaviors and opinions over time, addressing some of the limitations associated with cross-sectional surveys. We clarify the representativeness of using survey panel data in the methods section (line 267).

Methodological Transparency

The randomization process is well-described, but the manuscript would benefit from including the exact wording of all appeal conditions in the main text (not just supplemental materials).

Response: Thank you very much for the recommendation. We have included the exact wording of all appeal conditions for each experiment in the main text, as shown in the following figures:

- Fig2. Information Appeal Experiment in the Survey of Scientists’ Perceptions of Surveys
- Fig3. Information Appeal Experiment in the Vaccine Survey
- Fig4. Representation Appeal Experiment in the COVID-19 Survey Wave 2
- Fig5. Representation Appeal Experiment in the Public Trust in Science Survey
- Fig6. Representation Appeal Experiment in the Women’s Health Survey
- Fig7. Two-by-Two Experiment in the COVID-19 Survey Wave 4
- Fig8. Two-by-Three Experiment in the Science Impartiality Survey

To enhance brevity and readability, we have placed the full survey invitation letters, which contain additional administrative and consent-related information, in the supporting materials (S1 to S7 Appendix).

Response rates varied widely across surveys (e.g., 7.9% in COVID-19 Wave 4 vs. 44.3% in the Scientists’ Perceptions survey). Were there differences in survey length, timing, or incentives that could explain this?

Response: Thank you for raising this important point. The observed differences in response rates are primarily attributable to whether the survey sample includes non-SciOPS panel members. As discussed above, SciOPS panel members self-select into the panel and tend to exhibit higher levels of engagement and commitment to survey participation. Consequently, surveys composed solely of SciOPS panel members generally achieve significantly higher response rates.

As shown in Table 1, three surveys–(1) COVID-19 Survey Wave 2, (2) COVID-19 Survey Wave 4, and (3) the Public Trust in Science Survey—include both SciOPS and non-SciOPS panel members in their sample frames. These surveys exhibit overall response rates below 20%. Subgroup analyses further confirm that the response rates among non-SciOPS members are significantly lower than those among SciOPS panelists.

In contrast, the remaining surveys––(4) Survey of Scientists’ Perceptions of Surveys, (5) Women’s Health Survey, (6) Vaccine Survey, and (7) Science Impartiality Survey—sample only SciOPS panel members and report notably higher response rates, generally around 35%. We have updated Table 1 to reflect the overall response rates.

All surveys were similar in length, as detailed in Table 2, which presents the number of essential questions included in each. As shown in Appendices S1 to S7 (survey invitation letters), we informed participants that the surveys were brief, typically taking about 10 minutes to complete. No financial incentives were provided for participation in any of the surveys.

Regarding survey timing, surveys were administered throughout the calendar year, and we did not observe a consistent pattern of response rate variation attributable to timing. However, we did note evidence of survey fatigue in the COVID-19 Survey Wave 4, where the same sample frame yielded a lower response rate than in Wave 2.

We discuss this response rate distinction in the sampling section (line 303-306).

Practical Implications

The recommendations for tailoring appeals are useful but somewhat generic. Could the authors provide more specific guidelines (e.g., "For polarized topics, use self-representation appeals with mid-level detail")?

Response: Thank you for your comments. We have rewritten the practical implications section to include more detailed guidelines, as you suggested (line 605-614).

The finding that prior interaction with SciOPS diminishes the need for crafted appeals is intriguing. Is this a trust effect or familiarity effect? This could be explored further.

Response: Thank you for this comment. We agree that a further explanation about the familiarity effect helps improve our manuscript. We rewrote the corresponding text in the results and discussion sections to provide additional exploration of this point (line 490-499, 594-599).

**Minor Comments**

Clarity and Structure

The abstract could better highlight the mixed findings upfront (e.g., "Results show context-dependent effects...").

Response: Thanks for this constructive comment, and we agree it helps clarify the context-dependent effects in our results. We rewrote the abstract to highlight the mixed, context-dependent findings.

Table 2 and 3 are clear, but the significance markers (e.g., *p<0.1) could be better explained in the captions. Check for consistency in reporting p-values (e.g., p<0.1 vs. p<0.05) across tables.

Response: Thank you for your careful review and the comment. We double checked that the significance level markers (*p < 0.1, **p < 0.05, ***p < 0.01) are clearly explained in the table footnotes. They are now used consistently across all result tables from 2 to 7.

**Limitations**

The authors note that some nonrespondents may not have read the email content, but they could also discuss whether subject lines (uncontrolled in the experiments) might have influenced open rates.

Response: Thank you for this observation, and we agree that a deeper discussion incorporating subject line and email open rate benefits this study. We have revised the discussion section to further clarify this point (see lines 615–627). Specifically, while we acknowledge that some nonrespondents may have made their decision based solely on the subject line, we further note that representation appeal conditions were intentionally embedded in the subject line to ensure exposure regardless of whether or not the email is opened. Therefore, the concern for bias that subject line messages may bring to email open rates is minimal. In contrast, information appeal conditions were embedded only in the email body, as they could not be meaningfully conveyed in a brief subject line. While the unmanipulated subject line may have influenced the number of recipients who opened the email—and thus were exposed to the information appeal—this limitation does not bias the estimated effect of information appeal, as all experimental groups received the same subject line and email open rates can be assumed to be randomly distributed across experimental groups.

The potential for topic interest to confound results is acknowledged, but could be addressed statistically (e.g., by controlling for field-specific response rates).

Response: Thank you for the suggestion regarding use of academic field as a control. In all current logit models, we controlled academic fields. We further conducted a sensitivity check by examining field-specific response rates for both the Women’s Health Survey and the Vaccine Survey, under the assumption that faculty in public health may have greater interest in these topics. We found that public health faculty who received invitations with much information were significantly more likely to respond (15 percentage points higher, p < 0.1), compared to those receiving invitations with no information. However, beyond this subgroup, we found no other significant differences in the effectiveness of information or representation appeals across academic fields. We recommend that future research should consider developing direct measures of personal interest in the survey topic and examine how personal interest plays a key role in how recipients process and respond to survey requests. We also revised our discussion on this limitation (line 634-643).

Ethics and Data Availability

Ethical approval is noted, but the consent process (implied via email/survey initiation) could be briefly justified given the lack of written consent.

Response: Thank you for your comment and the opportunity to clarify our consent process. We have provided a brief justification in the revised Ethical Considerations section (lines 379-386) for the following reasons: (1) the use of implied consent is a common and accepted practice in online survey research, especially for minimal-risk studies; (2) studies have found no substantial difference in how well implied vs. written consent informs participants; (3) requiring signed consent can reduce survey response rates and deter participation; and (4) obtaining written consent is time-consuming and not feasible given the characteristics of our sample—busy academic scientists geographically distant from our institution.

Data availability is restricted due to privacy concerns, but could the authors share de-identified data or a codebook?

Response: Thank you for the comments on data availability. We share our de-identified dataset for the six experiments and replicable R code at Harvard Dataverse with the following reference: Chen, Tipeng, Timothy P. Johnson, Jinghuan Ma, Ashlee Frandell, Lesley Michalegko, and Eric W. Welch. 2025. “Replication Data for: Invitation Appeals and STEM Academic Scientists Research Participation: Findings from Six Survey Experiments.” Harvard Dataverse. https://doi.org/doi:10.7910/DVN/7YHUBI.

Improve readability by streamlining the methods section and adding a visual summary of key findings (e.g., a conceptual diagram).

Response: Thank you for the suggestions on improving readability. We streamline the methods section and summarize key findings in table 5 and in the discussion section.

Ensure all supplemental materials (e.g., S1 Appendix) are cited in the main text.

Response: Thank you for the comment. We have carefully reviewed the manuscript and confirmed that all the captions and in-text citations of supplemental materials are appropriately included in the main text in accordance with PLOS ONE guidelines.
